# The relationship of vitamin D deficiency and childhood diarrhea: a systematic review and meta-analysis

**DOI:** 10.1186/s12887-024-04599-0

**Published:** 2024-02-16

**Authors:** Glen Lazarus, I Gusti Ngurah Sanjaya Putra, Michelle Clarissa Junaidi, Jessica Sylvania Oswari, Hanifah Oswari

**Affiliations:** 1 Department of Child Health, Gastrohepatology Division, Cipto Mangunkusumo Hospital, Faculty of Medicine, Universitas Indonesia, Jakarta, Indonesia; 2 Department of Child Health, Gastroenterology and Hepatology Division, Medical School, Universitas Udayana, Bali, Indonesia

**Keywords:** Vitamin D deficiency, Pediatric, Childhood diarrhea

## Abstract

**Introduction:**

Vitamin D deficiency may increase the risk of childhood diarrhea. We aim to carry out a review and meta-analysis of the evidence relating vitamin D insufficiency to childhood diarrhea.

**Methods:**

We searched PubMed, Ovid, Scopus, and Cochrane Library (from inception to August 2022), then independently reviewed the eligibility, and read full-text reviews for selected articles. Keywords used were ‘vitamin D’, ’25-hydroxyvitamin D’, ‘vitamin D deficiency’, ‘diarrhea’, ‘gastroenteritis’, ‘children’, and ‘pediatric’. The search was limited to studies only in English and with available full-text. Year limitation was not applied in our search. Unpublished trials, dissertations, preliminary reports, conference abstracts, and repositories were excluded from the study. Newcastle-Ottawa Scale was used as the risk of bias assessment tool. Meta-analysis using the random-effects model was done.

**Results:**

Out of 5,565 articles, 12 articles were included in our systematic review, however only 7 articles were eligible for meta-analysis. Meta-analysis showed a statistically significant association between vitamin D deficiency and diarrhea in children in developing countries (OR = 1.79; 95% CI = 1.15 to 2.80; *p* = 0.01). On the secondary outcome, the association of vitamin D deficiency and duration or recurrences of diarrhea are conflicting.

**Conclusions:**

There is an association between vitamin D deficiency and the prevalence of diarrhea. Future studies should evaluate the causal association, the impact of vitamin D deficiency on the severity of diarrhea, and whether vitamin D deficiency treatments affects the prevalence of diarrhea.

**Supplementary Information:**

The online version contains supplementary material available at 10.1186/s12887-024-04599-0.

## Introduction

Bone health, calcium, and phosphorus homeostasis have been known to be influenced by vitamin D. However, data regarding extra-skeletal benefits are still limited [1]. The clinically measured vitamin D metabolite to evaluate vitamin D status is the serum 25-hydroxyvitamin D (25(OH)D) [2]. However, there are various targets and recommendations to maintain sufficient vitamin D levels. The definition of vitamin D deficiency (VDD) according to the American Academy of Pediatrics is 25(OH)D level of < 50 nmol/L (< 20 ng/mL) [3]. Additionally, the European Society for Paediatric Gastroenterology, Hepatology and Nutrition further defines a level of < 25 nmol/L as a severe deficiency [4]. Despite abundant sunlight, a cross-sectional study in Indonesia identified vitamin D insufficient-subjects in 47 of 120 school-aged children (39.17%), and two of these subjects were considered vitamin D deficient. For supplementation of vitamin D in all children, observational studies recommend a dosage of at least 10 µg/day [5].

The hallmark of diarrhea is an abrupt occurrence of stools that are watery and loose for three or more events per day. Primarily, acute diarrhea often occurs in children of < 5 years, with the most frequent cause being infections (viral, bacterial, and rarely parasitic) [6]. There are 2.5 billion diarrheal diseases in children of < 5 years globally, leading to an annual mortality rate of 1.8 million [7]. In children < 5 years, the prevalence of diarrhea, as reported by the Indonesian Ministry of Health, was 4 million cases or 17% in 2018 [8]. There is evidence that diarrhea may lead to malnutrition, growth disorders leading to stunting, and altered cognitive development, thus, affecting health and wellbeing for the children’s life [9].

The innate cell-mediated and adaptive immune response can be affected by micronutrients. One of these micronutrients is vitamin D, considering its ability to inhibit the proliferation of B cells and block its differentiation and immunoglobulin secretion, suppress the proliferation of T cells, and inhibit dendritic cell differentiation and maturation. Vitamin D potentially plays a role in preventing diarrhea in childhood [10]. Furthermore, emerging evidence has shown to support vitamin D in repairing the colonic epithelium and providing integrity [11]. Also, a statistically significant association between acute bacterial diarrhea and vitamin D concentration has been proven in past studies [9]. Therefore, to lower the burden of infectious diseases such as diarrhea, maintaining sufficient vitamin D levels might be a public health measure that is also low-cost [12]. In addition, various studies have also shown the association between infectious and inflammatory diseases such as thoracic empyema, otitis media, pulmonary tuberculosis, asthma, and low vitamin D levels. Nevertheless, the association between diarrhea and vitamin D levels is still rarely reported in studies [11]. There has been no systematic review done regarding this topic. Therefore, we aim to conduct a systematic review and meta-analysis regarding the association between vitamin D deficiency and childhood diarrhea to provide new insight and possible solutions to better health outcomes.

## Methods

We conducted this study following the Preferred Reporting Items for Systematic Reviews and Meta-Analyses flow diagram (PRISMA) guidelines [13].

### Types of studies

Observational studies with cohort and cross-sectional study designs that determine the correlation between serum vitamin D status in children with diarrhea were included. Studies that only reported levels of serum vitamin D (without classifying the patients) were excluded.

### Types of participants

Children 0–18 years of age were included. We excluded studies with no full-text and/or using a language other than English. Studies that involved diarrhea due to non-infection causes, such as irritable bowel syndrome (IBS), inflammatory bowel disease (IBD), and/or autoimmune-related diarrhea were also excluded from this study.

### Type of intervention and control

Serum vitamin D levels are determined by laboratory measurement using enzyme-linked immunosorbent assay (ELISA) or chemiluminescence assay (CLIA) on blood samples reported as ng/mL. Other possible units reported will be converted to ng/mL for better interpretation and comparison. We included studies involving pediatric patients with diarrhea or gastroenteritis.

### Types of outcomes

The primary outcome is the prevalence of diarrhea in the vitamin D deficiency and control group reported as number of diarrhea cases and/or odds ratios. The secondary outcomes are the number of diarrhea episodes (reported as number of recurrences per period of time or as a rate, for example days per child per year) and duration of diarrhea (reported as hours or days) in the vitamin D deficiency and control group.

### Search strategy

The following databases: PubMed, Ovid, Scopus, and Cochrane Library (from inception to August 2022) were searched thoroughly. The keywords used include ‘vitamin D’, ’25-hydroxyvitamin D’, ‘vitamin D deficiency’, ‘diarrhea’, ‘gastroenteritis’, ‘children’, and ‘pediatric’. Further searches were done from the reference lists of included studies. No year limitation was applied in our search. Unpublished trials, dissertations, preliminary reports, conference abstracts, and repositories were excluded from the study. The Supplementary Table 1 shows the search terms for PubMed.

### Selection of studies

Titles and abstracts of each database were independently screened by two researchers (GL and MCJ). Full-text review and eligibility review were conducted independently for selected articles. We consulted with a senior author (HO) for any disagreements met during the discussion.

### Data extraction

Data extraction includes author, year of publication, country, study design, sample, parameters measured, and results. We independently extracted and input all data into the Microsoft Excel spreadsheet. Compared results and differences were resolved by discussion or, if required, a consultation with a senior author of the review team (HO).

### Study risk of bias assessment

The Newcastle-Ottawa Scale (NOS), comprising three major components (selection, comparability, exposure/outcome) with eight small items and scores of 0–9, was used to assess the methodological quality of observational and non-randomized studies [15]. Two authors assessed the included studies, and a senior author was involved until a consensus was reached when two authors disagreed.

### Synthesis methods

Meta-analysis was conducted using the Review Manager (RevMan) Software version 5.4 from Cochrane Collaboration with random effects setting. We assessed the heterogeneity among studies using the *χ* [2] test, and we calculated the *I* [2] to estimate the amount of variation. If we had to find the potential source of heterogeneity, subgroup analyses would be conducted.

## Results

**Fig. 1:**
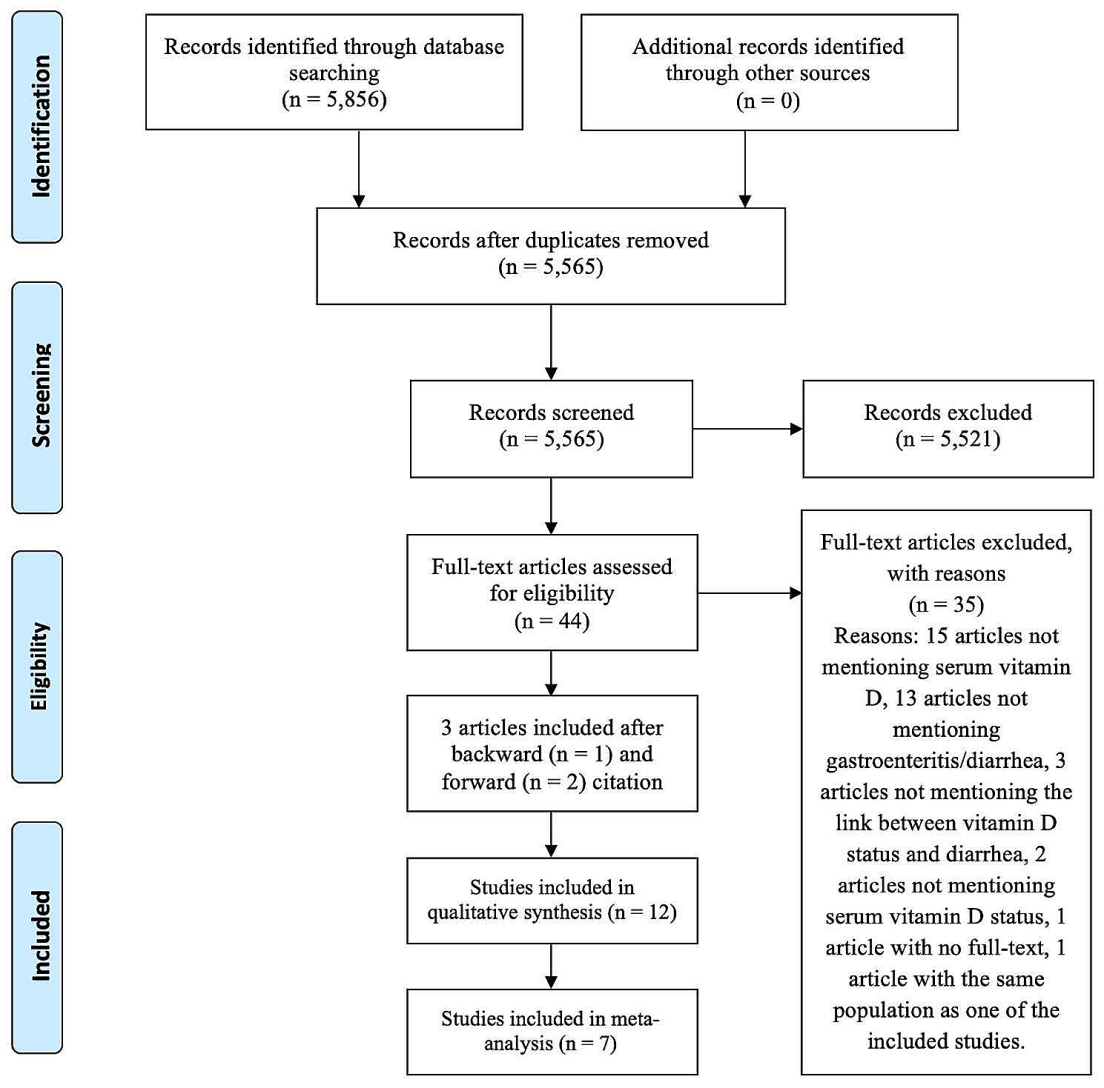
PRISMA flow diagram

Figure 1 shows the summary of the PRISMA flow diagram with the selection process. Initially, we obtained 5,856 studies from three databases and excluded 291 duplicated articles. We excluded 5,521 articles because the titles and abstracts did not contain data regarding serum vitamin D and childhood diarrhea, were review articles, and/or had no available full text. We further excluded 35 articles after reviewing the full text because they did not mention serum vitamin D or gastroenteritis/diarrhea, had no analysis between vitamin D status and diarrhea, not mentioning vitamin D status, or had no full-text. We also exclude one article by Ahmed et al. as these two studies were not removed as duplicates in the initial step because of their different titles. However, on further investigation we found that both studies have the same populations. Consequently, we included 12 studies for this systematic review [10–12, 14–22]. Table 1 display the characteristics of studies included in this meta-analysis.

**Table 1:** Characteristics of included studies

Author, Date, Country	Design	Age of Children	Vitamin D Status Definition (Case Group)	Outcome	Results	NOS
Bener et al., 2009, Qatar [14]	CS	0–15 years old	Deficiency (< 20 ng/mL)	Presence of gastroenteritis	Case 92/315 (29.2%) Control 27/143 (18.89%)	7
Binks et al., 2014, Australia [15]	CC	< 3 years old	Insufficiency (< 30 ng/mL)	Hospitalised with gastroenteritis	Case 12/24 (50%) Control 8/50 (16%)	7
Bucak et al., 2016, Turkey [16]	PS	< 48 months old	Deficiency (< 20 ng/mL)	Rotaviral diarrhea	Case 59/63 (93.65%) Control 11/74 (14.86%)	8
Chowdhury et al., 2017, India [17]	CS	6–30 months old	Deficiency (< 10 ng/mL)	Prevalence of diarrhea	Case 17/331 (5.14%) Control 32/629 (5.09%)	8
Hassam et al., 2019, Tanzania [11]	CC	< 5 years old	Deficiency (< 20 ng/mL)	Prevalence of diarrhea	Case 20/35 (57.14%) Control 27/59 (45.76%)	8
Mahyar et al., 2019, Iran [10]	CS	2 months – 12 years old	Deficiency (< 20 ng/mL)	Acute bacterial diarrhea	Case 30/53 (56.6%) Control 30/67 (44.78%)	8
Talachian et al., 2015, Iran [12]	CS	6 months – 15 years old	Deficiency (< 20 ng/mL)	Acute infectious diarrhea	Case 9/9 (100%) Control 16/41 (39.02%)	8
Abed et al., 2014, Egypt [18]	CS	4–12 years old	Deficiency (< 20 ng/mL)	Duration and recurrence of diarrhea	See Table 2	8
Ahmed et al., 2016, Australia [19]	CC	6–24 months old	Deficiency (< 20 ng/mL)	Number of diarrhea episode	See Table 2	9
Basaran et al., 2022, Turkey [20]	RS	6 months – 6 years old	Deficiency (< 20 ng/mL)	Rotaviral diarrhea	See Table 2	8
Sudfeld et al., 2017, US [21]	PC	6 weeks – 6 months old	Deficiency (< 20 ng/mL)	Mean diarrhea diagnoses/year	See Table 2	9
Thornton et al., 2013, Colombia [22]	CS	5–12 years old	Deficiency (< 20 ng/mL)	Diarrhea rate per child-year	See Table 2	9

Half of the included studies are cross-sectional (*n* = 6), three case-controls [11, 15, 19], two prospective studies [16, 21], and one retrospective study [20]. Half of the included studies were conducted in Asia, two studies in Africa [11, 18], two studies in Australia [15, 19], and one study each from North America [21] and South America [22]. They were all published between 2009 and 2022.

We performed the quality assessment of twelve studies using the Newcastle-Ottawa Scale (NOS). Table 1 shows scores ranging from 7 to 9 for all included studies. A score of 7–9 may be deemed as study of high quality, although a standardized criterion for score interpretation has not yet been made. We provided Supplementary Table 2 for a detailed table for NOS assessment.

We did not include five studies in the meta-analysis for primary outcome because of different reported outcomes, and we summarized the findings in Table 2.

**Table 2:** Results summary of studies not included in meta-analysis

Author, Year, Country	Summary
Abed et al., 2014, Egypt [18]	- Duration of diarrhea in VDD group is 2.77 ± 0.73 days, Vitamin D insufficient is 2.67 ± 0.65 days, and Vitamin D sufficient group is 2.85 ± 0.69 days (*p* = 0.82) - Number of diarrheal recurrences in VDD group is 5.71 ± 2.23, Vitamin D insufficient is 6.0 ± 1.91, and Vitamin D sufficient group is 3.31 ± 1.49 days (*p* = 0.035)
Ahmed et al., 2016, Australia [19]	- In normal-weight children, diarrheal episode in VDD group is 368, Vitamin D insufficient is 337, and Vitamin D sufficient group is 121.
Basaran et al., 2022, Turkey [20]	- The number of patients with VDD was significantly higher in the rotaviral group than in the control group (after excluding those with an allergic disease).
Sudfeld et al., 2017, US [21]	- There was no statistically significant association between vitamin D status with the incidence of diarrhea (after multivariate analyses).
Thornton et al., 2013, Colombia [22]	- Patients with VDD had higher rates of diarrhea with vomiting than vitamin D sufficient group. - VDD group had twice as many days with diarrhea and vomiting (after adjusting the patient’s age, sex, and socioeconomic status) (*p* = 0.009).

### Primary outcomes

**Fig. 2:**
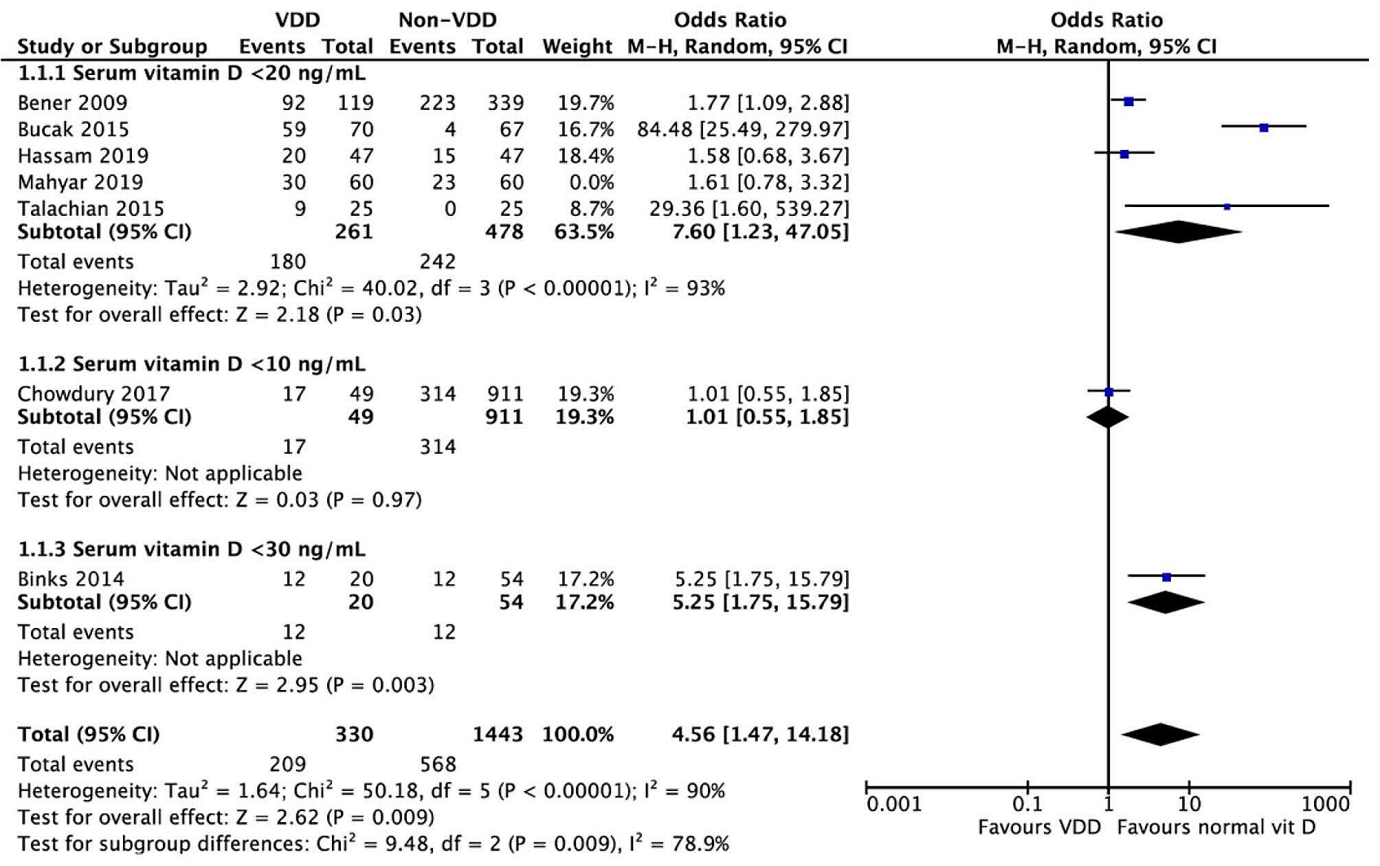
Forest plot with subgroup analysis according to VDD definition

**Fig. 3:**
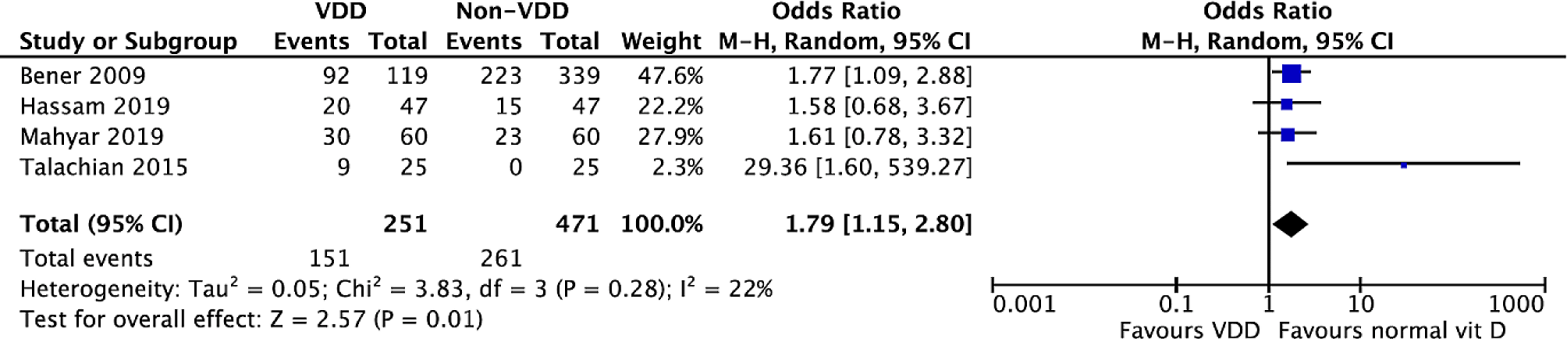
Forest plot for meta-analysis of diarrhea prevalence in VDD group (< 20 ng/mL) in developing countries

The meta-analysis investigating the compared prevalence of diarrhea in VDD and the control group is shown in Fig. 2. There was a higher prevalence of diarrhea in the VDD group (OR = 3.73; 95% CI = 1.47 to 9.46; *p* = 0.006) but high heterogeneity among the studies (*I*^2^ = 88%, *p* < 0.00001). A subgroup analysis separating studies using below 20 ng/mL as their VDD definition [10–12, 16, 18] still showed a high heterogeneity (*I*^2^ = 90%, *p* = 0.01) with a higher prevalence of diarrhea prevalence in the VDD group (OR = 5.04; 95% CI = 1.37 to 18.47; *p* = 0.01) (See Fig. 2). Figure 3 shows that the prevalence of diarrhea in developing countries is higher in the VDD group (serum vitamin D < 20 ng/mL) (OR = 1.79; 95% CI = 1.15 to 2.80; *p* = 0.01) with low heterogeneity among the studies (*I*^2^ = 22%, *p* = 0.28).

### Secondary outcomes

Two studies described diarrhea duration in the VDD group vs. the control group. A study by Abed et al. [18]. reported that the duration of diarrhea was not statistically different between those groups, while a study by Thornton et al. [22] showed the duration of diarrhea and vomiting were doubled in patients with VDD compared to the control group, with a significant *p* value.

Three studies reported conflicting results in diarrhea episodes/recurrences between the VDD group vs. control group. A study by Ahmed et al. [19]. reported that the VDD group has more episodes than those with vitamin D sufficient status, but with no *p* value reported. Another study by Abed et al. [18] reported that patients with VDD have more recurrences than those with sufficient vitamin D status with a statistically significant *p* value. One study by Chowdhury et al. [17]. reported that diarrhea episodes had no statistically significant association with vitamin D status.

## Discussion

VDD and diarrhea are two of the most common diseases affecting pediatric population globally, particularly in developing countries [23, 24]. There has been no systematic review examining the possible link between VDD and childhood diarrhea. Our meta-analysis and systematic review aim to assess the association between these two diseases.

We included 12 studies in this systematic review. Initial analysis showed a higher prevalence of diarrhea in the VDD group (OR = 3.73; 95% CI = 1.47 to 9.46; *p* = 0.006) with high heterogeneity (*I*^*2*^ = 88%, *p* < 0.00001). Therefore, we conducted a subgroup analysis separating studies that were conducted in developing countries. Out of four articles, we found that there was a significantly higher prevalence of diarrhea in the VDD group in developing countries (OR = 1.79; 95% CI = 1.15 to 2.80; *p* = 0.01) [10–12, 14]. This significant association is found in various types and settings of diarrhea, including acute gastroenteritis in general, rotaviral diarrhea, acute bacterial diarrhea, and acute gastroenteritis in hospitalized children. Five studies provide information for the secondary outcome of this study. These five studies are not included in the meta-analysis of primary outcome because outcomes from these studies are in parametric terms, while our primary outcome is the prevalence or absence of diarrhea in VDD compared to control. Out of the five studies, two described diarrhea duration, while three reported diarrhea episodes or recurrences between the VDD vs. control group. The association regarding VDD and diarrhea’s duration and episodes/recurrences are still conflicting. These differences might be caused by several factors such as different VDD criteria used, nutritional status, exposure to sunlight, sample size, and socioeconomic factors.

Typically, Vitamin D regulates gene transcription from the vitamin D receptor (VDR). Vitamin D’s role in the immune system includes inhibiting Th17 and Th1 responses, promoting T-regs, impairing the development of B cell and its function, and stimulating antimicrobial peptides from immune cells. In recent literature, lower vitamin D has been reported to correlate with infectious disease occurrences (respiratory tract, asthma, and viral infections) [25]. Vitamin D’s role as an antimicrobial is based on its antibacterial peptides, including cathelicidin, *β*-defensin, and lysozyme, and increased intestinal epithelial macrophage activity. Cathelicidin have antiviral and antifungal properties and it is able to form transmembrane pores in the cell wall of bacteria [26]. In addition, luminal bacteria are constantly exposed to epithelial cells and gastrointestinal tracts’ lamina propria macrophages. This has been known to have an essential role in developing normal intestine and innate immunity. Furthermore, Paneth cells of the intestine are able to produce antimicrobial peptides regulated by VDR signaling [27, 28]. Therefore, the defensive effect of vitamin D in bacterial diarrhea can be defined by these mechanisms, which increase resistance against invading pathogenic organisms in the intestine, including *Shigella* and *Salmonella*. Similarly, animal studies have shown that reduced *Salmonella* invasion is associated with VDR expression, and anti-bactericidal effects on *E. coli* have been found in vitamin D-regulated antimicrobial peptides [10]. In cases of rotavirus diarrhea, the mechanisms of vitamin D protection have only been suggested in animal studies, presumably involving *retinoic acid-inducible gene-1* (RIG-1). This study revealed that vitamin D-supplemented porcine has a better food intake, larger body weight, higher intestinal villi, and lower concentration of IL-2, IL-6, and interferon-β [25]. Moreover, significant associations have been found between the microbiome composition and vitamin D. Many studies have also found that the signaling of vitamin D3/VDR can modulate the quantity and distribution of tight junction protein; therefore, decreasing gut permeability and preventing bacterial translocation [26]. 

Our analysis adds that vitamin D deficiency should be considered as a comorbid that can be present in children with diarrhea in developing countries; thereby focused interventions on both entities should be considered. Children with diarrhea or gastroenteritis warrants further investigation regarding their vitamin D status. This approach hopefully could optimize the management of both diseases. Our study has several weaknesses, including that there was no cohort study included. Therefore, we could not conclude whether VDD and diarrhea are affected by similar factors or if there are causative and/or direct associations. In addition, viral, bacterial, or non-infectious diarrhea occurs through different mechanisms; however, we did not specify the causative cause of acute diarrhea. Furthermore, we did not include studies with chronic diarrhea, including irritable bowel syndrome (IBS), inflammatory bowel disease (IBD), and/or autoimmune-related diarrhea. In future studies, we suggest investigating the causal association of diarrhea and vitamin D deficiency of cohort studies, vitamin D status impact on the severity of diarrhea, recurrence, or duration, and whether VDD intervention can reduce the diarrhea prevalence and/or severity.

## Conclusions

Our systematic review and meta-analysis found an association between VDD and diarrhea in children living in developing countries. Future studies should evaluate the causal association using cohort design studies, the impact of VDD towards the severity of diarrhea, and whether VDD treatment can help to reduce diarrhea.

### Electronic supplementary material

Below is the link to the electronic supplementary material.

**Additional files 1**: **Supplementary Table 1**. PubMed Search Terms.

**Additional files 2**: **Supplementary Table 2**. Newcastle-Ottawa Scale (NOS) assessment of included studies.

## Data Availability

All data generated or analysed during this study are included in this paper (and its supplementary information files).

## Declarations

### Risk of publication bias

The exclusion of studies in which full texts are not available and non-English studies can introduce potential publication bias in this study as important research conducted in other languages with less positive or inconclusive results may have been omitted.

## Abbreviations

VDD: Vitamin D deficiency
25(OH)D: Serum 25-hydroxyvitamin D
VDR: Vitamin D receptor
ELISA: Enzyme-linked immunosorbent assay
CLIA: Chemiluminescence assay
NOS: Newcastle-Ottawa Quality Assessment Scale
PRISMA: Preferred Reporting Items for Systematic Reviews and Meta-Analyses flow diagram
RIG-1: Retinoic acid-inducible gene-1
IBS: Irritable bowel syndrome
IBD: Inflammatory bowel disease
