# Supplementary material for: The relationship of vitamin D deficiency and childhood diarrhea: a systematic review and meta-analysis
Source: BMC Pediatr. 2024 Feb 16;24:125. doi: 10.1186/s12887-024-04599-0 (PMC10870643; doi:10.1186/s12887-024-04599-0)
Supplement: Supplementary file 1 — Additional files 1: Supplementary Table 1. PubMed Search Terms. [file 12887_2024_4599_MOESM1_ESM.docx]

**Supplementary Table 1. PubMed Search Terms**

| ("vitamin d"[MeSH Terms] OR "vitamin d"[All Fields] OR "ergocalciferols"[MeSH Terms] OR "ergocalciferols"[All Fields] OR ("25 hydroxyvitamin d"[Supplementary Concept] OR "25 hydroxyvitamin d"[All Fields] OR "25 hydroxyvitamin d"[All Fields] OR "calcifediol"[MeSH Terms] OR "calcifediol"[All Fields]) OR ("vitamin d deficiency"[MeSH Terms] OR "vitamin d deficiency"[All Fields])) AND ("diarrhea"[MeSH Terms] OR "diarrhea"[All Fields] OR "diarrheas"[All Fields] OR "diarrhoea"[All Fields] OR "diarrhoeas"[All Fields] OR ("gastroenteric"[All Fields] OR "gastroenteritis"[MeSH Terms] OR "gastroenteritis"[All Fields] OR "gastroenteritides"[All Fields])) AND ("child"[MeSH Terms] OR "child"[All Fields] OR "children"[All Fields] OR "child s"[All Fields] OR "children s"[All Fields] OR "childrens"[All Fields] OR "childs"[All Fields] OR ("paediatrics"[All Fields] OR "pediatrics"[MeSH Terms] OR "pediatrics"[All Fields] OR "paediatric"[All Fields] OR "pediatric"[All Fields]))  **Search Engine: PubMed**  **Search Date: August 17^th^ , 2022**  **Articles Identified: 292** |
| --- |
