# Supplementary material for: The relationship of vitamin D deficiency and childhood diarrhea: a systematic review and meta-analysis
Source: BMC Pediatr. 2024 Feb 16;24:125. doi: 10.1186/s12887-024-04599-0 (PMC10870643; doi:10.1186/s12887-024-04599-0)
Supplement: Supplementary file 2 — Additional files 2: Supplementary Table 2. Newcastle-Ottawa Scale (NOS) assessment of included studies. [file 12887_2024_4599_MOESM2_ESM.docx]

**Supplementary Table 2. Newcastle-Ottawa Scale (NOS) assessment of included studies.**

|  | *Selection* | *Comparability* | *Exposure* |
| --- | --- | --- | --- |
| Bener *et al*., 2009^1^ | Gastroenteritis definition is not provided. All eligible cases with gastroenteritis are included. Controls were taken from the community and had no history of the disease. **(3/4)** | This study controls for same age group between case & control groups, but not other factors (other diseases). **(1/2)** | Exposure was determined by a structured survey and assessment. This study used a same method of ascertainment for cases and controls, and same non-response rate for both groups. **(3/3)** |
| Binks *et al.*, 2014^2^ | Gastroenteritis was determined by ICD-10 codes. All eligible cases with gastroenteritis are included. Controls were taken from the hospital and had no history of the disease. **(3/4)** | This study controls for same age group between case & control groups, but not other factors (other diseases). **(1/2)** | Exposure was determined by a secure record. This study used a same method of ascertainment for cases and controls, and same non-response rate for both groups. **(3/3)** |
| Bucak *et al*., 2016^3^ | Rotaviral diarrhea was determined by laboratory. All eligible cases are included. Controls were taken from the hospital and had no history of the disease. **(3/4)** | This study controls for same age group between case & control groups, and other factors. **(2/2)** | Exposure was determined by a secure record. This study used a same method of ascertainment for cases and controls, and same non-response rate for both groups. **(3/3)** |
| Chowdhury *et al*., 2017^4^ | Diarrhea was determined clinically. All eligible cases with diarrhea are included. Controls were taken from the community and had no history of the disease. **(4/4)** | This study controls for same age group between case & control groups, but not other factors (other diseases). **(1/2)** | Exposure was determined by a structured survey. This study used a same method of ascertainment for cases and controls, and same non-response rate for both groups. **(3/3)** |
| Hassam *et al*., 2019^5^ | Diarrhea was determined clinically. All eligible cases with diarrhea are included. Controls were taken from the hospital and had no history of the disease. **(3/4)** | This study controls for same age group between case & control groups, and other factors. **(2/2)** | Exposure was determined by a secure record. This study used a same method of ascertainment for cases and controls, and same non-response rate for both groups. **(3/3)** |
| Mahyar *et al*., 2019 ^6^ | Bacterial diarrhea was determined by laboratory examination. All eligible cases with bacterial diarrhea are included. Controls were taken from the hospital and had no history of the disease. **(3/4)** | This study controls for same age group between case & control groups, and other factors. **(2/2)** | Exposure was determined by a secure record. This study used a same method of ascertainment for cases and controls, and same non-response rate for both groups. **(3/3)** |
| Talachian *et al*., 2015^7^ | Acute infectious diarrhea was determined clinically according to WHO definition. All eligible cases with diarrhea are included. Controls were taken from the hospital and had no history of the disease. **(3/4)** | This study controls for same age group between case & control groups, and other factors. **(2/2)** | Exposure was determined by a secure record. This study used a same method of ascertainment for cases and controls, and same non-response rate for both groups. **(3/3)** |
| Abed *et al*., 2014 | Recurrent diarrhea was determined clinically. All eligible cases with recurrent diarrhea are included. Controls had no history of the disease but no description of their source (hospital or community). **(3/4)** | This study controls for same age group between case & control groups, and other factors. **(2/2)** | Exposure was determined by a secure record. This study used a same method of ascertainment for cases and controls, and same non-response rate for both groups. **(3/3)** |
| Ahmed *et al*., 2016^8^ | Diarrhea was determined clinically, while vitamin D deficiency was determined by laboratory examination. All eligible cases are included. Controls were taken from the community and had no history of the disease. **(4/4)** | This study controls for same age group between case & control groups, and other factors. **(2/2)** | Cases were taken from medical record only. This study used a same method of ascertainment for cases and controls, and same non-response rate for both groups. **(3/3)** |
| Basaran *et al.*, 2022^9^ | Rotaviral diarrhea was determined clinical and laboratory examination, while vitamin D deficiency was determined by laboratory examination. All eligible cases are included. Controls were taken from the hospital and had no history of the disease. **(3/4)** | This study controls for same age group between case & control groups, and other factors. **(2/2)** | Cases were taken from medical record only. This study used a same method of ascertainment for cases and controls, and same non-response rate for both groups. **(3/3)** |
| Sudfeld *et al.*, 2017^10^ | Vitamin D deficiency was determined by laboratory examination. All eligible cases are included. Controls were taken from the community and demonstrated that the outcome of interest was not present at start of study. **(4/4)** | This study controls for same age group between case & control groups, and other factors. **(2/2)** | Cases were taken from medical record only. This study used a same method of ascertainment for cases and controls, and same non-response rate for both groups. **(3/3)** |
| Thornton *et al*., 2013^11^ | Vitamin D deficiency was determined by laboratory examination. All eligible cases are included. Controls were taken from the community and demonstrated that the outcome of interest was not present at start of study. **(4/4)** | This study controls for same age group between case & control groups, and other factors. **(2/2)** | Cases were taken from medical record only. This study used a same method of ascertainment for cases and controls, and same non-response rate for both groups. **(3/3)** |
